# Syndromic Approach to Arboviral Diagnostics for Global Travelers as a Basis for Infectious Disease Surveillance

**DOI:** 10.1371/journal.pntd.0004073

**Published:** 2015-09-15

**Authors:** Natalie B. Cleton, Chantal B. E. M. Reusken, Jiri F. P. Wagenaar, Elske E. van der Vaart, Johan Reimerink, Annemiek A. van der Eijk, Marion P. G. Koopmans

**Affiliations:** 1 Erasmus Medical Centre, Rotterdam, The Netherlands; 2 National Institute for Public Health and Environment (RIVM), Bilthoven, The Netherlands; 3 University of Reading, Reading, Berkshire, United Kingdom; Emory University, UNITED STATES

## Abstract

**Background:**

Arboviruses have overlapping geographical distributions and can cause symptoms that coincide with more common infections. Therefore, arbovirus infections are often neglected by travel diagnostics. Here, we assessed the potential of syndrome-based approaches for diagnosis and surveillance of neglected arboviral diseases in returning travelers.

**Method:**

To map the patients high at risk of missed clinical arboviral infections we compared the quantity of all arboviral diagnostic requests by physicians in the Netherlands, from 2009 through 2013, with a literature-based assessment of the travelers’ likely exposure to an arbovirus.

**Results:**

2153 patients, with travel and clinical history were evaluated. The diagnostic assay for dengue virus (DENV) was the most commonly requested (86%). Of travelers returning from Southeast Asia with symptoms compatible with chikungunya virus (CHIKV), only 55% were tested. For travelers in Europe, arbovirus diagnostics were rarely requested. Over all, diagnostics for most arboviruses were requested only on severe clinical presentation.

**Conclusion:**

Travel destination and syndrome were used inconsistently for triage of diagnostics, likely resulting in vast under-diagnosis of arboviral infections of public health significance. This study shows the need for more awareness among physicians and standardization of syndromic diagnostic algorithms.

## Author Summary

Physicians attending travelers with particular symptoms often neglect those infections that are transmitted by arthropods like ticks and mosquitoes (arboviruses) or don’t test for the appropriate arboviruses. This is because arboviruses cause symptoms that are similar to more common infections and because there is a geographical overlap in the arbovirus infections that people have a large chance of being infected with. We compared the amount of times that Dutch physicians had patients tested for arboviral infections with the likelihood that Dutch travelers would be exposed to particular arboviruses. Whereas research and diagnostics generally focus on only one virus, our study was uniquely comprehensive and systematic in that it analyzed multiple viruses simultaneously on the basis of a unique national database. The research shows that the current viruses travelers are tested for is incomplete and likely many more people carry these kinds of diseases than are diagnosed. As these diseases pose potential public health threats, physicians should be more aware of the diseases that travelers could be infected with, and protocols are needed regarding what infectious diseases physicians should check for when patients present with particular symptoms.

## Introduction

Globalization has resulted in a steep increase in travel and trade.[1, 2] In recent decades it has contributed to the spread of diseases that traditionally emerged only regionally but now threaten populations across the globe, stressing the need for global health surveillance.[1, 2] Among these emerging threats, arboviruses form a unique group, with a large public health impact in endemic countries, a tendency to expand their geographical distribution through trade and travelers, and colonize previously unaffected areas. Due to their vector-borne and often zoonotic nature, they require targeted surveillance and control schemes. This requirement is particularly relevant when evaluating symptoms of illness in travelers. Of all those returning from developing, tropical, or subtropical countries, 8% require medical care on return.[3] For those returning from Africa and Southeast Asia, fever is the most common reason for seeking medical care; for travelers returning from the Caribbean and South America, rash is the most common reason. Around 50% of the cases remain undiagnosed in clinics focused on travel medicine, and this percentage is likely higher in less specialized clinics.[3] The traveler’s personal physician is therefore an important link in ongoing arbovirus surveillance in travelers and the gate-keepers of disease detection.

Correct diagnosis of arbovirus infections in travelers is challenging. Arboviruses have overlapping geographical distributions and cause symptoms that coincide with more common infections.[4] If general practitioners consider an arbovirus infection in their differential diagnosis, they commonly test for the best known arboviral disease, Dengue virus (DENV). Laboratory diagnostics for travelers are largely based on serologic testing, since viremia is short-lived and has often already dropped to undetectable levels when severe symptoms appear and diagnostics are performed.[5, 6] The use of serologic results for arbovirus diagnosis and surveillance requires careful evaluation due to cross-reactivity of antibodies to related viruses.[7] Also, several vaccines, notably for Yellow fever, Tick-borne encephalitis and Japanese encephalitis, can cause false-positive serological tests.[7]

For these reasons, arbovirus illness is under-diagnosed, as evidenced by studies of unexplained illness in returned travelers.[8–10] A potential solution would be the development of syndromic arboviral disease detection methods that cover the most common arboviruses and simultaneously provide surveillance information.[11] Here we aimed to assess the potential added value of syndrome-based approaches for diagnosis and surveillance of neglected arboviral diseases in returning Dutch travelers.

## Methods

**Fig 1:**
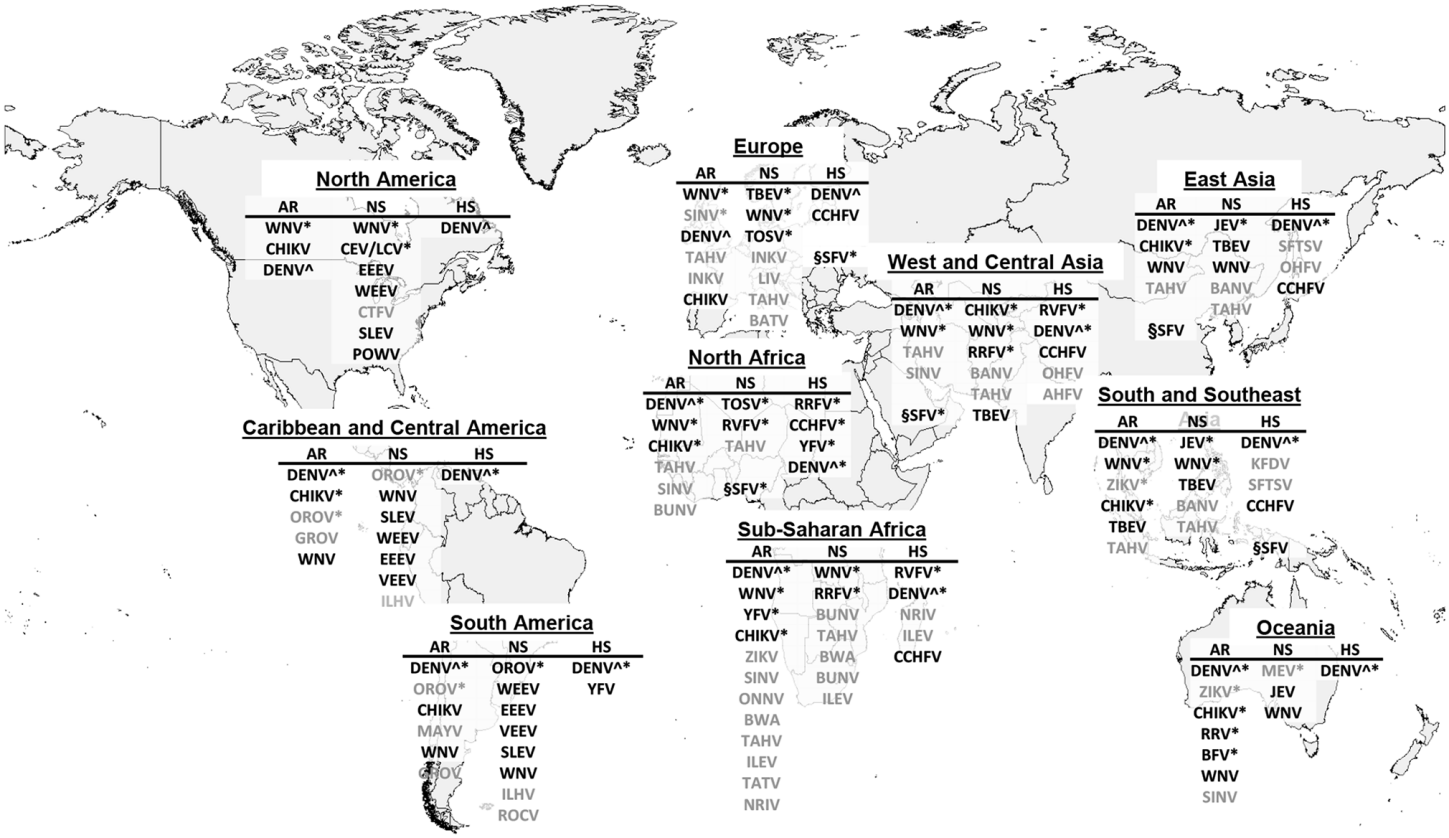
Geographical distribution of medically important arboviruses that cause febrile disease in humans. All arboviruses cause febrile symptoms, but symptoms more specific to certain viruses are represented in three columns: 1) Arthralgia-Rash (AR); 2) Neurological symptoms (NS), and 3) Hemorrhagic symptoms (HS). Arboviruses not known to cause more than febrile symptoms are preceded with a §-sign. Arboviruses more likely to be diagnosed in travelers are followed by *. DENV^ is a serocomplex encompassing multiple dengue viruses that can cause similar clinical disease in humans. For viruses in gray type, diagnostics are unavailable in the Netherlands but in most cases can tested through the European Network for Imported Viral Diseases (ENVID). Geographical regions based on UN definitions of world regions. EU, Sub-Saharan Africa and South & Southeast Asia regions are grouped in these representations for visual clarity but are subdivided according to UN definitions for analysis as can been seen in Figs 3–6. AR = arthralgia-rash; NS = neurological symptoms; HS = hemorrhagic symptoms; AKHV = Alkhurma hemorrhagic fever virus; BANV = Banna virus; BFV = Barmah Forest virus; BWAV = Bwamba virus; BUNV = Bunyamwera virus; CEV = California encephalitis virus; CHIKV = Chikungunya virus; CTFV = Colorado tick fever virus; CCHFV = Crimean-Congo hemorrhagic fever; DENV = Dengue virus; EEEV = Eastern equine encephalitis virus; GROV = Guaroa virus; ILEV = Ilesha virus; ILHV = Ilheus virus; JEV = Japanese encephalitis virus; KFDV = Kyasanur Forest disease virus; LCV = La cross virus; LIV = Louping Ill virus; MAYV = Mayaro virus; MURV = Murray Valley virus; NRIV = Ngari virus; OHFV = Omsk hemorrhagic fever virus; ONNV = O’Nyong Nyong virus; OROV = Oropouche virus; RVFV = Rift Valley fever virus; ROCV = Rocio virus; RRV = Ross river virus; SFV = Sandfly fever (Naples / Sicilian / other); SFTS V = Severe Fever with Thrombocytopenia Syndrome Virus; SINV = Sindbis virus; SLEV = St. Louis encephalitis virus; TAHV = Tahyna virus; TATV = Tataguine virus; TBEV = Tick-borne encephalitis virus; TOSV = Toscana virus; VEEV = Venezuelan equine encephalitis virus; WEEV = Western equine encephalitis virus; WNV = West Nile virus; YFV = Yellow fever virus; ZIKV = Zika virus.

To map the patients high at risk of missed clinical arboviral infections in returned Dutch travelers, we compared the quantity and quality of all arboviral diagnostic requests by Dutch physicians, from 2009 through 2013, with a previously extensive literature-based assessment of travelers’ likely infection with an arbovirus.[4] The overlapping syndromes and geography, based on and updated from that review are depicted in Fig 1.

### Database construction

For retrospective patient analysis, a database was created by integrating data from the two arbovirus diagnostic reference centers in the Netherlands: Erasmus Medical Centre in Rotterdam and The National Institute for Public Health and the Environment in Bilthoven. Previously, we described trends of DENV diagnostics in the Netherlands from 2000–2010.[12] The current study included almost all arbovirus diagnostic requests from Dutch physicians from 2009 through 2013 in the Netherlands. In the case of DENV not all data was included because 10% of the DENV diagnostics were performed outside the arbovirus reference centers and were not included in this dataset. For syndromic analysis, only entries were included where travel and clinical history were provided. To define the syndromes, entries in the database were reviewed by a consultant microbiologist, and infectious disease clinicians assigned them to syndrome categories (Table 1).

**Table 1:** Clinical manifestations classified per syndrome used for search in diagnostic database. Search was based on approximation of listed terms in multiple languages.

Syndrome	Respiratory	Enteric	Febrile	Neurological	Skin	Rheumatic	Hemorrhagic
**Symptoms**	Throat ache	Diarrhea	Fever	Glasgow coma scale	Rash	Rheumatic pain	Secondary/ primary hemostasis
	Coughing	Vomiting	Pyrexia	Coma	Exanthema	Joint pain	Hematemesis
	Wheezing	Dehydration	Febrile	Reduced responsiveness	Spots	Arthralgia	Hemoptysis
	Hoarseness	Nausea	Temperature	Epileptic symptoms	Erythema	Arthritis	Melena
	Nasal / ocular discharge	Gastroenteritis	Malaise	Encephalitis	Maculopuritis	Vasculitis	Hemorrhagic diatheses
	Bronchitis	Abdominal pain	Flu-like symptoms	Meningitis			Thrombocytopenia
	Pneumonia			Myelitis			Petechial
	Rhinitis			Ataxia			Ecchymosis
	Hypoxia			Paresis			Anemia
	Dyspnea			Flaccid paralysis			DIC (diffuse intra-vascular coagulation)
	Apnea			Neurological symptoms			Reduced clotting
	Pleural effusion			Neurological dysfunction			Reduced platelet count
	Chest congestion			Neurological disease			
	Earache			Polyradiculitis			
	Otalgia						
	Sinusitis						
	Epiglottitis						

### Patient test result classification

Due to the laboratory-specific variety in diagnostic methods used, we classified each patient’s test results according to the validated methods and cut-offs for the pertinent laboratory. Results were classified as positive for a disease if the patient had (1) a positive PCR result with <40 cycles, (2) an IgM above an individual laboratory-determined cut-off, or (3) a minimum fourfold increase in IgG titers between two consecutive samples. For DENV patients, (4) a positive non-structural protein 1(NSI) antigen-capture test was among the criteria.[6]

### Analysis of the likelihood of arboviral infections in travelers

The likelihood of infections by arboviruses other than DENV was based on a previously published article in which we developed syndromic diagnostic algorithms based on data from an exhaustive review of the literature addressing geographic distribution and prevalence of arboviruses by syndrome.[12] Optimal diagnostic algorithms using a combination of clinical syndromes and geographical distribution presented were updated and used as a basis for our current analysis (Fig 1). In short, criteria used to prioritize arboviruses for the diagnostic algorithm were: a) circulation in urbanized areas, due to the use of humans as reservoir hosts, or the presence of reservoir hosts colonizing urban areas, b) known endemic disease, c) tourist activity in the area, d) high rate of exposure in resident population, and e) recorded cases of infections in travelers.[4] These diagnostic algorithms were used in the current article to identify gaps that may occur with a physician-indexed single-virus approach.

### Travel data

Travel data for Dutch travelers was based on the year 2011. They were extracted from a commercial database “ContinuVakantieOnderzoek” (CVO) created for trend analysis in the tourism industry. Its data are collected and converted into national numbers every three months by interviewing individuals in about 15,000 Dutch households on their travel destinations, activities, lodging, transport, and booking method.[13] Using data from 2011 provided a representative distribution of Dutch travel behavior from 2009–2011. Only slight country specific fluctuations were reported.[13]

### Statistical analysis

The analysis was performed in STATA.[14] Pearson's chi-square test was used to assess for equality of proportions. Multivariable logistic regression models (Table 2) reporting odds ratios were used with a 95% confidence level.[14] Heatmaps were generated using the additional R package “stats”[15] and based on pair-wise correlation between rows and columns.

**Table 2:** Adjusted odds ratios of statistically significant predictive syndromes for a positive test outcome. The test is stated in column 1, with corresponding variables in column 2. Variables were adjusted for age, sex, travel region, and diagnostic laboratory.

Dependent variable	Independent Variable	Adjusted Odds ratio	95% CI	P-value
DENV-positive versus negative test outcome (based on 1843 patients with DENV-diagnostic tests performed)	Febrile symptoms	2.0	1.2–3.0	*<0*.*01*
	Rash	1.9	1.3–2.5	*<0*.*01*
	Arthralgia-arthritis	0.5	0.3–0.8	*<0*.*01*
	Hemorrhagic symptoms	2.8	1.8–4.5	*<0*.*01*
	Neurological symptoms	0.7	0.2–1.9	*0*.*4*
	Respiratory symptoms	0.5	0.3–0.8	*<0*.*01*
	Enteric symptoms	0.8	0.5–1.1	*0*.*1*
CHIKV-positive versus negative test outcome (based on 736 patients with CHIKV-diagnostic tests performed)	Febrile symptoms	1.5	0.7–3.2	*0*.*3*
	Rash	4.0	2.2–7.1	*<0*.*01*
	Arthralgia-arthritis	2.9	1.7–5.2	*<0*.*01*
	Hemorrhagic symptoms	0.4	0.1–3.4	*0*.*4*
	Neurological symptoms	0.7	0.1–6.2	*0*.*7*
	Respiratory symptoms	0.3	0.1–1.1	*0*.*1*
	Enteric symptoms	0.4	0.1–1.1	*0*.*1*

### Ethical statement

This research was conducted in accordance with the Dutch law on medical research (WMO), article 1. In compliance with Dutch Law and medical ethical guidelines, no personal identifiers were included and no informed consent was required for use of data in this study.

## Results

### General dataset

Over the five year study period 8126 patients were tested for arboviral diseases in the Netherlands. Of the patients, 44% presented to larger hospitals or specialized travel clinics. All other patients were seen at smaller hospitals or local clinics. Molecular tests comprised 1.3% of diagnostic tests performed. Larger hospitals and specialized travel/tropical clinics tested on average for 1.7 viruses per patient compared to 1.2 in smaller hospitals and local clinics. The patient male to female ratio was 1.04. Vaccination history was recorded on the diagnostic request for only 14 patients (<1%).

Of all patients, 2153 (26%) had information on travel history and clinical history and were thus included for further syndrome and travel-based analysis. Of these, 23% had provided a second serum sample needed for determination of a potential IgG titer increase. With a median of 7 days, the average number of days elapsed between onset of symptoms and first sampling was 17.5 (95%CI 14.0–20.3). This number is based on the 317 patients with clinical and travel history for whom this chronological information was recorded. Elapsed time did not differ between patients seen at specialized hospitals/clinics and those visiting smaller hospitals/clinics.

### Comparison travel destination

**Fig 2:**
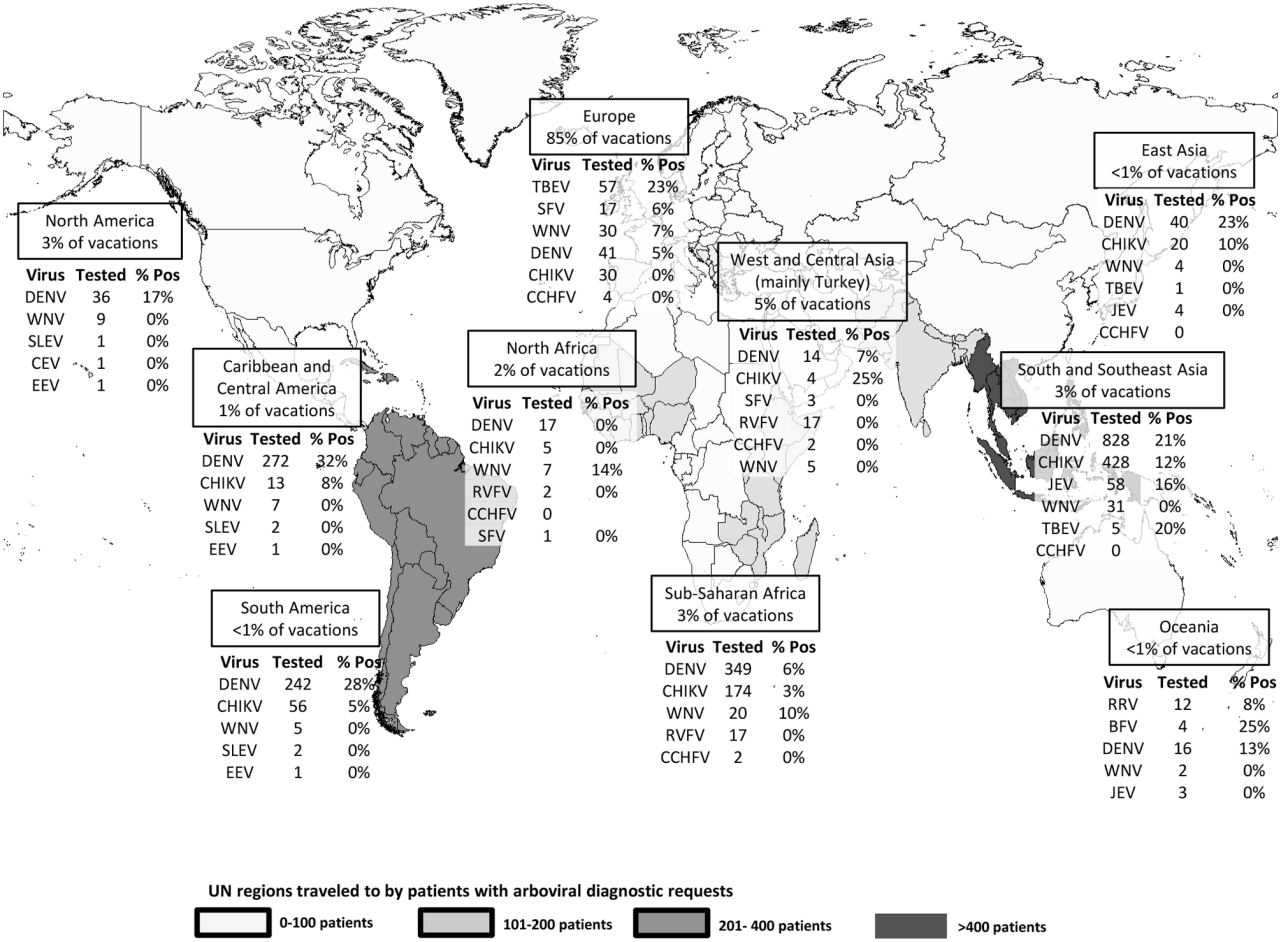
Geographical depiction of the number of diagnostic tests requested after travel to each region (see gray shading and tables) from 2009 to 2013. Boxes show number and percentage of all vacations booked from the Netherlands to each region in 2011.

We analyzed the travel data of Dutch travelers in 2011 to determine the range and importance of arbovirus tests needed to cover the differential diagnosis for travelers with illness after return from the various destinations. In 2011, approximately 84% of Dutch travelers traveling abroad stayed within Europe. Western Asia (predominantly Turkey) was the most popular non-European destination, with nearly one million Dutch vacations booked annually (Fig 2).[13] The most diagnostic requests (35%) by far, however, were for travelers returning from destinations in South and Southeast Asia, while only 3% of all travelers had this region as their destination.

### Diagnostic requests and outcomes per region

The number of diagnostic requests by travel region and the proportion of positive test results (Fig 2) show that DENV testing was by far the most commonly requested (86%), yielding the highest absolute number of cases (Fig 2). When comparing the numbers of requests and proportions of positives by region of travel, substantial differences were observed: diagnostic requests for ill travelers returning from sub-Saharan Africa were frequent but not often positive, whereas ill travelers returning from popular arbovirus-endemic regions in Central and Western Asia were rarely tested. A low number of patients who had traveled within Europe were tested. DENV was tested (N = 41) almost as often as tick-borne encephalitis virus (TBEV) (N = 57), for which exposure is far more likely. Of note, two of these European travelers tested DENV-positive. One was a tourist returning from Croatia, who tested DENV-IgM-positive and borderline NS1-positive. The other tourist had taken a five-day trip to Southern France and was DENV IgM- and NS1-positive 10 days after return. However, 14 days previous to onset of symptoms, this traveler had been in Thailand before traveling on to France. Another virus considered endemic to Europe is Sindbis virus (SINV), for which diagnostics are not readily available in the Netherlands. Nor are they available for oropouche virus (OROV), endemic to South America.

### Syndromes reported

**Fig 3:**
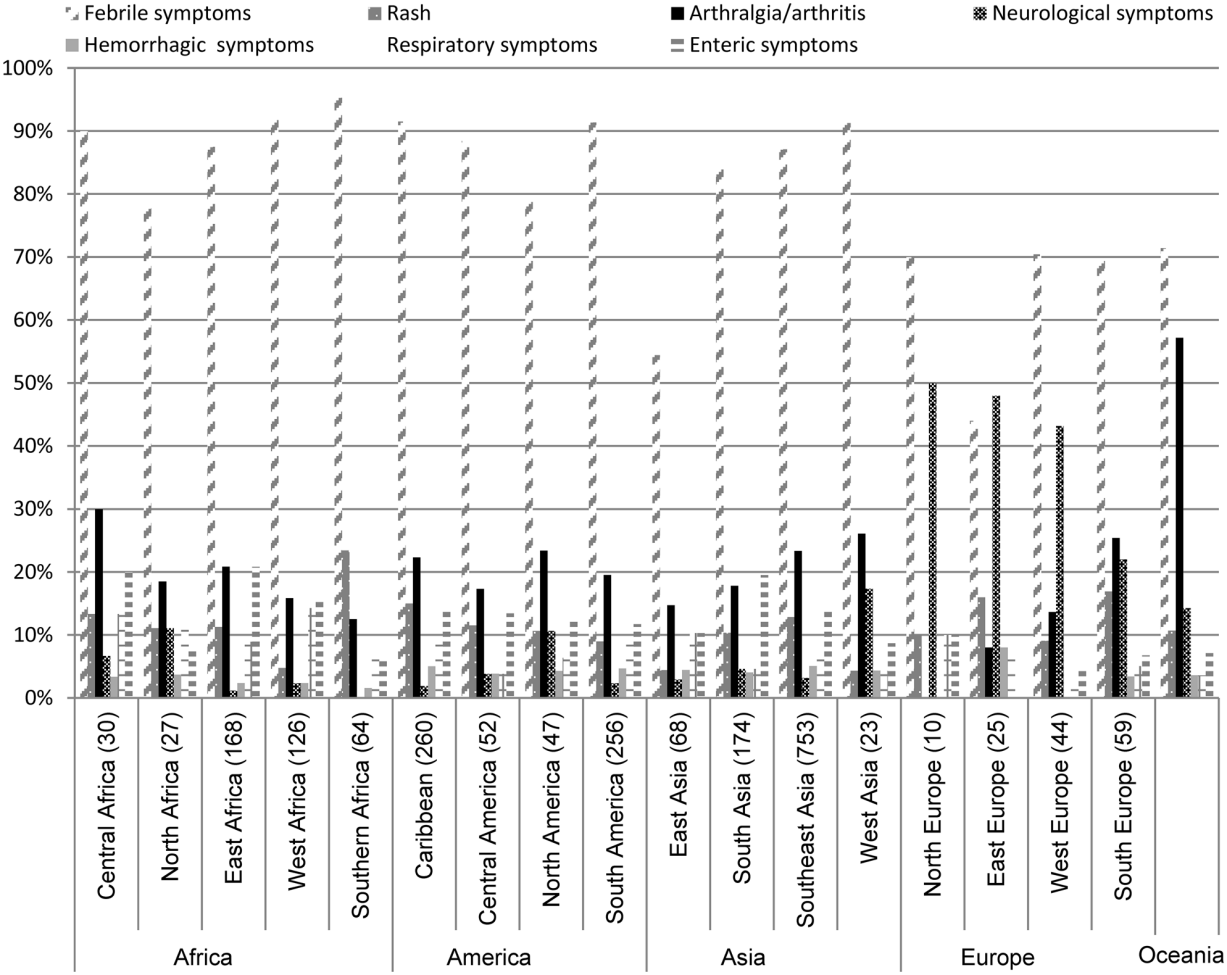
Percentage of patients (left axis) with arbovirus diagnostic requests presenting with symptoms by travel destination (horizontal axis). The number of patients per group is shown in parentheses on the horizontal axis (based on 2153 patients with both travel and clinical history).

To assess the potential use of diagnostic requests for syndrome surveillance by region, we analyzed the symptoms recorded for each patient returning from a particular travel destination. Nearly all patients (86%) reported fever, followed by arthralgia/arthritis (22%) and enteric symptoms (14%). Information divided per travel region showed regional variation in symptoms recorded (Fig 3). For all regions, fever was the most reported symptom. Proportionally, neurological symptoms were more often reported for travelers returning from a European destination than for travelers from other regions. Arthralgia-arthritis was recorded more frequently for travelers returning from Oceania, with rash being most recorded for Southern Africa compared to other regions.

### Comparing diagnostic requests to diagnostic algorithms

**Fig 4:**
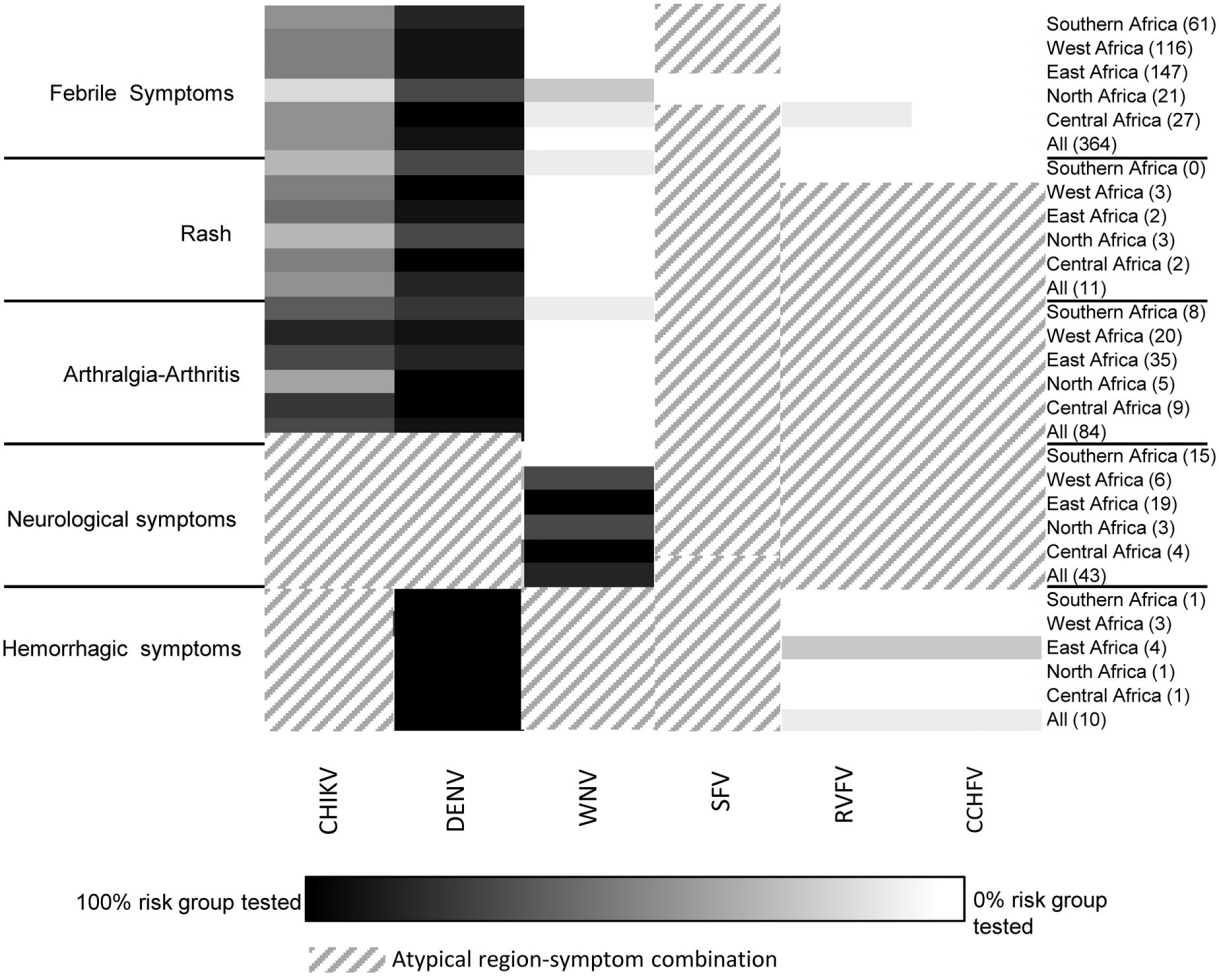
Heatmapsshowing percentage of patients with a travel history to Africa, divided by region (right axis) and recorded symptoms (left axis), who were tested for each arbovirus (horizontal axes) posing a risk on that continent (see Fig 1). The number of patients in each region-symptom combination follows each region in parentheses, far right. Groups in which a 100% of patients with a specific region-symptom combination were tested are depicted as black, with a sliding scale to white for groups in which 0% of patients were tested. Region-symptom combinations that are atypical for a certain arbovirus are depicted as diagonal lines.

**Fig 5:**
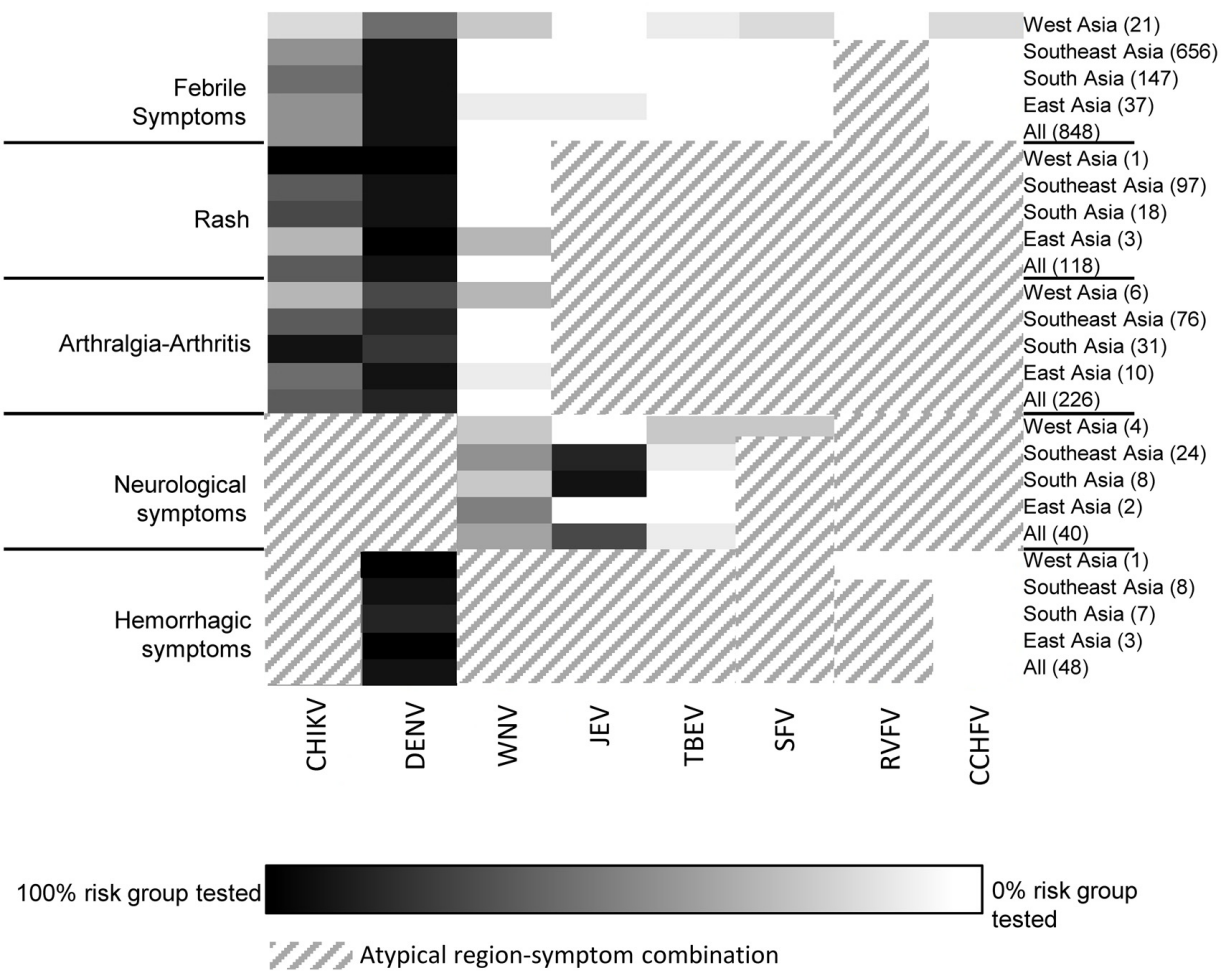
Heatmap showing percentage of patients with a travel history to Asia, divided by region (right axis) and recorded symptoms (left axis), who were tested for each arbovirus (horizontal axes) posing a risk on that continent (see Fig 1). The number of patients in each region-symptom combination follows each region in parentheses, far right. Groups in which a 100% of patients with a specific region-symptom combination were tested are depicted as black, with a sliding scale to white for groups in which 0% of patients were tested. Region-symptom combinations that are atypical for a certain arbovirus are depicted as diagonal lines.

**Fig 6:**
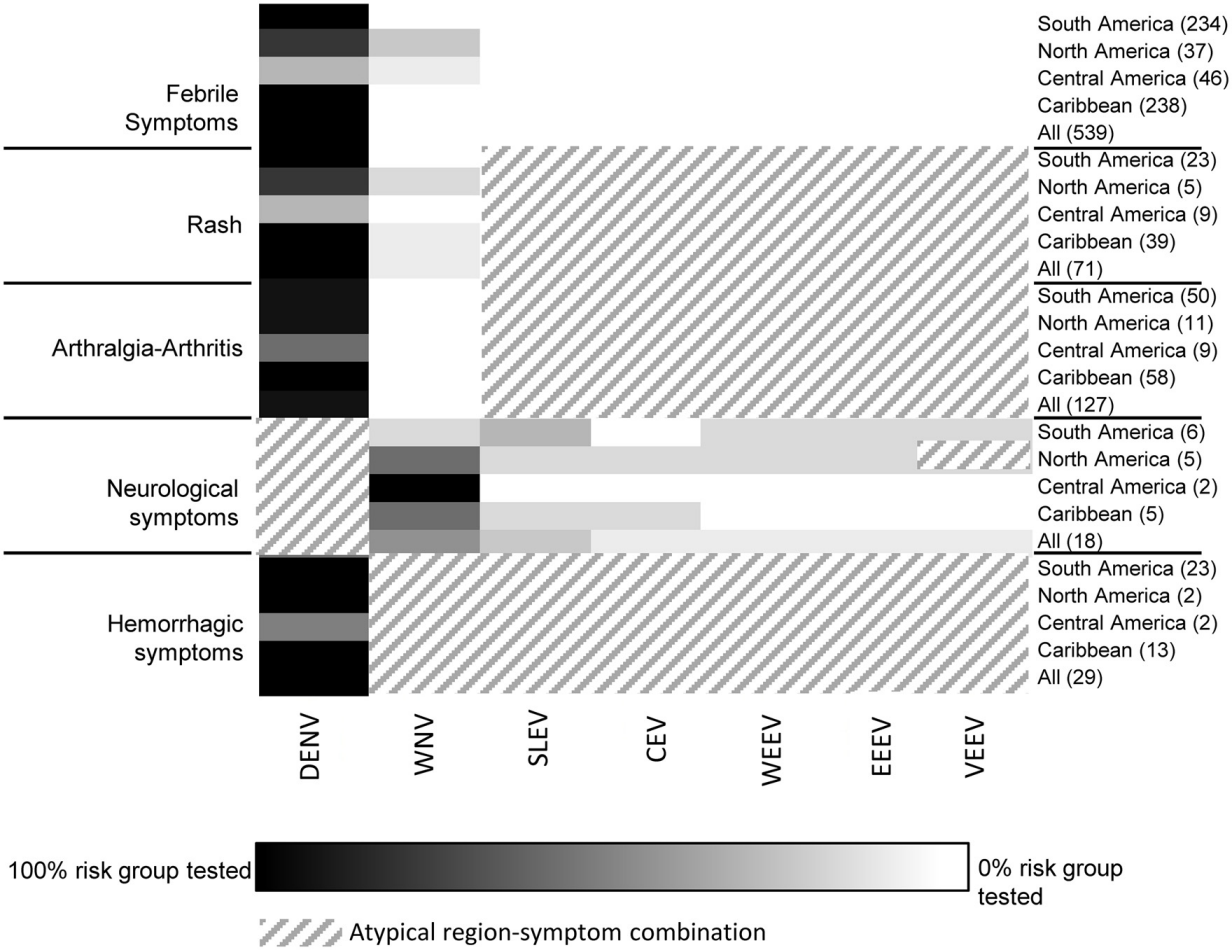
Heatmap showing percentage of patients with a travel history to Americas, divided by region (right axis) and recorded symptoms (left axis), who were tested for each arbovirus (horizontal axes) posing a risk on that continent (see Fig 1). The number of patients in each region-symptom combination follows each region in parentheses, far right. Groups in which a 100% of patients with a specific region-symptom combination were tested are depicted as black, with a sliding scale to white for groups in which 0% of patients were tested. Region-symptom combinations that are atypical for a certain arbovirus are depicted as diagonal lines.

Three heatmaps were created to visualize per continent (Africa, Asia and the Americas) the correlation between the physicians’ diagnostic requests and the literature-based syndromic algorithms (Fig 1). In the heatmaps, diagnostic requests are grouped based on the clinically important arboviral diseases per region within each continent (Figs 4–6). For most regions, Dutch physicians requested DENV diagnostics for 100% of the travelers who had recorded symptoms corresponding to DENV infection (fever, rash and joint pain). For some regions, a lower percentage of such patients was tested, i.e. Northern Africa (67%) (Fig 4), Western Asia (57%) (Fig 5) and Central America (38%) (Fig 6). In all regions, CHIKV testing was less frequently requested than DENV testing, even though the infections overlap in geographical distribution and range of symptoms to a great extent. On average, 45% of patients with febrile symptoms, rash and/or arthralgia after travel to CHIKV-risk areas in Asia were not tested for CHIKV. Patients with symptoms suggesting West Nile Virus (WNV), Japanese encephalitis (JEV), Rift Valley fever virus (RVFV) and TBEV were tested infrequently (0 to 25%) and only in association with neurological symptoms. Diagnostics on all other viruses presented in Figs 4–6 were minimally requested.

### Predictive factors for positive tests

We analyzed the association between symptoms recorded and test outcomes for DENV and CHIKV requests in Dutch travelers (Table 2). Patients with rash, hemorrhagic symptoms and fever had an increased odds of testing positive for DENV, but respiratory symptoms decreased the odds of being DENV-positive (OR 0·5). Positive test outcomes for CHIKV were associated with arthralgia combined with rash. Both DENV and CHIKV were positively associated with travel history to Southeast Asia.

## Discussion

Here we assessed the extent of missed arboviral infections in travelers by a retrospective database analysis of all arboviral diagnostic requests in the Netherlands, from 2009–2013, in comparison with a literature-based assessment of arbovirus exposure while traveling (Fig 1). We found clear evidence for patient groups high at risk of being under-diagnosed for arboviral disease when evaluated by syndrome and by region. While DENV diagnostics are routinely requested, other relevant arboviruses are neglected, in particular CHIKV. Arthralgia, for example, is not only associated with DENV infections but also with many arboviruses, including CHIKV, as we found when calculating odds ratios within the current Dutch data.[4] Nevertheless, less than 55% of patients with symptoms compatible with CHIKV infection were tested (Fig 4a–4c). Interestingly, hemorrhagic symptoms and rash have a much higher odds ratio than arthralgia-arthritis for diagnosing DENV. Although arthralgia is an important symptom in dengue patients, rash and fever are often more pronounced.[16] In the case CHIKV arthralgia-arthritis is more pronounced and is known to have a higher predictive value for distinguishing CHIKV from DENV in endemic settings.[16, 17] Additionally, CHIKV is less well known by physicians in non-endemic countries so might be only considered if DENV diagnostics are negative.

The analysis of diagnostic requests by region showed a bias toward the more well-known arboviral risk areas such as Southeast Asia (Figs 2 and 3). For travelers within Europe, arbovirus diagnostics are rarely requested, despite high incidence rates of TBEV reported across Europe and continuing circulation of WNV in parts of Europe popular with Dutch tourists.[8, 18] This is a general trend also seen in previous reports on travel associated infection presenting in Europe.[19] Housing type and location during travel is an import risk factor for exposure to specific vectors,[20, 21] and outdoor camping is popular among travelers in Europe.[13] The number of CHIKV and DENV requests within Europe was almost equivalent to the number of TBEV and WNV test requests, while only a small number of CHIKV and DENV have been reported.[22–24] The low number of TBEV and WNV requests may reflect a lack of physician awareness of European arboviruses and their risk to travelers; it may also reflect financial restrictions or limited time.[18]

Our analysis showed that physicians were more likely to extend the diagnostic panel for patients with more severe or very specific symptoms. For instance, diagnostics for WNV and Western equine encephalitis virus (WEEV) were usually requested only for patients with neurological complaints, even though fever is the most common clinical presentation in >90% of WNV and WEEV patients.[25] Similarly, RVFV diagnostic requests were limited to patients with hemorrhagic symptoms (HS) and neurological symptoms (NS), although these severe symptoms occur in less than 1% of cases, and most patients present only with febrile symptoms (Fig 4a–4c).[26] This bias toward severe symptoms was likewise reflected by the finding that patients referred to large hospitals and travel clinics were more extensively evaluated than those visiting small hospitals and local clinics. Reasons for this difference were not assessed in our study but are likely related to the fact that 1) general practitioners often omit arbovirus diagnostics, due in part to budgetary constraints; 2) they may lack knowledge on arboviral disease, and 3) may believe that an arbovirus diagnosis is unlikely to influence their treatment decisions, particularly if symptoms are mild. However, even mild arbovirus infections can eventually cause severe or chronic symptoms like arthralgia and, in any case, they pose a potential risk to health workers. Lack of proper diagnosis may lead to unnecessary complications or extensive later testing of patients. A possible solution to this problem is diagnostic centers providing syndromic and region based diagnostic packages for travelers as presented by the algorithms here.[4] These can be continuously updated in collaboration with specialized physicians and Public Health professionals. This will relieve the general physicians from keeping up to date on such a complex and continuously changing area. At the same time physicians are provided with a complete diagnostic selection and data are more suitable for use in surveillance.

Our results show a large variation in the timing of first diagnostic sampling. In our study, 50% of travelers contacted a healthcare provider during the first week of illness. This means that 50% did not, and viremic patients may introduce viruses into a region, when appropriate vectors are available, [23, 27] or pose a risk for nosocomial infection.[28, 29] Only 1.3% of all diagnostic tests performed were molecular, while 50% of patients fell within the range advised for molecular testing. The timeframe for molecular and serological diagnostics overlap to a great extent. Within the first days of illness, however, serology has a low sensitivity.[6] A number of the DENV cases may have been secondary, tertiary or quaternary infections. This reduces the sensitivity of serological detection by IgM in non-primary infections significantly.[6] Many patients are therefore probably missed due to lack of molecular testing within this timeframe. To use diagnostic data for syndromic surveillance, a two-tiered approach could be employed. First, samples collected after three days of illness onset would provide syndromic information by multiplex serologic testing. Second, if testing showed increased circulation of a target virus, confirmation and genomic surveillance would follow in patients suspected to harbor that virus sampled within seven days of illness.

There are a number of limitations to this study. Nearly all patients tested for arboviral diseases in the five-year-period in the Netherlands were included. This group, however, only consists of patients that seek medical attention after travel and that are suspected of an arboviral infection by a clinician. Asymptomatic patients and patients where clinicians did not consider an arboviral disease are missed. Almost all patients lack vaccination history. Patients with recent yellow fever vaccinations could cause positive false positive serological tests.[6] Lack in reporting vaccination history and the resulting possible flavivirus cross-reactivity due to vaccination are known problems when using flavivirus serological diagnostic data.[9] Both diagnostic centers had extensively validated tests internally with yellow fever vaccines and changed diagnostic cut-offs provided by manufacturer to compensate if possible. However, false positive tests due to vaccination cannot be excluded.

Infectious disease diagnostics and surveillance of travelers is primarily focused on those cases or diagnostic outcomes selected and reported by physicians.[30–33] Although this approach provides essential information, many patients remain undiagnosed, and re-evaluation of the selected pathogens has been advised.[10, 31, 34] However, much knowledge on probable arbovirus exposure of travelers is based on information originating from the destination country, which may have limited surveillance and diagnostic capabilities. In some of these countries, large-scale surveillance projects using a more syndromic approach to infectious diseases have shown extensive under-diagnosis and under-recognition of the importance of many arbovirus diseases as a cause of common syndromes.[35, 36] This underlines the need, in the Netherlands and other affluent countries, for more systematic syndrome-based diagnosis and surveillance in travelers to these regions. It demonstrates the added value of using routine travel information to support national and international surveillance programs. For such surveillance, capturing only a fraction of all cases may still provide reliable information on disease trends and possibly local outbreaks, provided the selection is systematic.[9] It is also important in terms of preparedness for emerging infectious diseases.

### Conclusion

A physician’s diagnostic requests for returned travelers can play a key role in infectious disease surveillance. However, while travel destination and syndrome could be used for triage and diagnostics, such use is inconsistent. We found clear evidence of patient groups at risk of under-diagnosis of arboviral disease when evaluated by syndrome and by region.

Based on a comparison between all arboviral diagnostic requests by physicians in the Netherlands between 2009 and 2013 with a literature-based assessment of the likely exposure of the patients to an arbovirus, we showed that while dengue virus diagnostics are routinely requested, other relevant arboviruses such as chikungunya virus are neglected, even if travelers present with relevant symptoms and return from countries where the viruses are endemic. We also showed that for travelers to European destinations, arbovirus diagnostics were rarely requested and that for almost all arboviruses and travel destinations, diagnostics were requested only when patients presented with severe symptoms.

Whether the low number of requests and overemphasis of physicians on patients presenting with severe symptoms reflects a lack of physician awareness of arboviruses and their risk to travelers, financial restrictions or limited time, it points at possible gaps in preparedness. Our paper shows that in order to limit the amount of missed clinical arboviral infections, and to increase the level of awareness of arboviral infections of public health significance, physicians should rely on diagnostics and surveillance with a syndromic approach and matching laboratory methods.
